# Effect of heat-killed *Lacticaseibacillus paracasei* KW3110 on mild to moderate seasonal allergic rhinitis symptoms in Japanese adults: a randomized, double-blind, placebo-controlled parallel-group study

**DOI:** 10.3389/fnut.2025.1568329

**Published:** 2025-06-25

**Authors:** Yoshihiko Sugihara, Jiawen Zheng, Yuko Fukushima, Hajime Nozawa, Yusuke Ushida, Ryohei Tsuji, Yoshitaka Okamoto, Daisuke Fujiwara

**Affiliations:** ^1^Institute of Health Sciences, Kirin Holdings Co., Ltd., Fujisawa, Japan; ^2^Chiba Rosai Hospital, Chiba, Japan

**Keywords:** *Lacticaseibacillus paracasei* KW3110, lactic acid bacteria, allergic rhinitis, probiotics, cedar pollen

## Abstract

**Background:**

In recent years, the prevalence of seasonal allergic rhinitis has increased rapidly in Japan, becoming a social problem. Therefore, we evaluated the effect of tablets containing *Lacticaseibacillus paracasei* KW3110 (*L. paracasei* KW3110) on allergic symptoms in the nose and eyes.

**Methods:**

In a randomized, double-blind, placebo-controlled, parallel-group study, 120 healthy adults with mild to moderate allergic rhinitis ingested either a placebo tablet or a test tablet containing *L. paracasei* KW3110 for 12 weeks (from January to April). Subjects who did not regularly use medication and experienced nasal and eye discomfort in early spring were selected. Allergic symptoms in the nose and eyes were assessed using subjective symptom surveys and diagnosis by an otorhinolaryngologist.

**Results:**

Compared with the placebo group, the *L. paracasei* KW3110 group showed a significant improvement in the score of “nasal symptoms” (*p* < 0.05) and “outdoor activities” (*p* < 0.05) in the Japanese Rhino-conjunctivitis Quality of Life Questionnaire (JRQLQ No.1) at 4 weeks when pollen dispersal begins, and “runny nose” (*p* < 0.05) in a diary questionnaire on nasal and eye symptoms at 8 weeks. Diagnosis by an otorhinolaryngologist showed a significant decrease in the score of “paroxysmal sneezing or rhinorrhea” (*p* < 0.05) and “classification of severity of allergic rhinitis symptoms” (*p* < 0.05) at 4 weeks, and the number of subjects scoring “worse” (*p* < 0.05) in the efficacy assessment.

**Conclusion:**

These findings suggest that intake of *L. paracasei* KW3110 is effective in alleviating nasal symptoms in subjects with mild to moderate allergic rhinitis, which can be useful for preventing allergic rhinitis without medication.

**Clinical trial registration:**

https://www.umin.ac.jp/, identifier UMIN000053239.

## 1 Introduction

In recent years, the number of patients with allergic diseases has been steadily increasing around the world. In the epidemiological Survey of Allergic Rhinitis in Japan 2019, the prevalence of allergic rhinitis was 49.2% (1), making it a serious social problem with approximately one in two people suffering from allergic rhinitis. Furthermore, the overall prevalence of pollinosis was 42.5%, and the prevalence of cedar pollen allergy, the most common type of pollinosis, was 38.3%. Efforts to combat pollinosis are important; it was confirmed that the prevalence of cedar pollen allergy has increased by approximately 10% over the past 10 years, and it is possible that the prevalence of pollinosis will increase further in the future (1). Allergic rhinitis is a type I inflammatory disease of the nasal mucosa caused by allergens such as pollen, mites or house dusts from environment. The main symptoms are paroxysmal reactions such as recurrent sneezing, watery rhinorrhea, and nasal blockage (2). These symptoms can reduce the quality of life (QOL) on a day-to-day basis. Treatment for allergic rhinitis includes drug therapy using antihistamines and steroids, immunotherapy, and surgery. Drug therapy accounts for a large proportion of treatments (3); however, it can cause side effects such as drowsiness, which can interfere with daily life. The causes of allergic rhinitis are divided into genetic factors and environmental factors such as allergens. Rapid changes in the living environment caused by progressive industrial and scientific technologies and global warming, which drives rising temperatures, have increased air pollutants and other allergens (4). While the reduction in opportunities for exposure to microorganisms due to improvements in sanitary conditions (the hygiene hypothesis) could exacerbate allergic rhinitis (5). It has been reported that reduced exposure to microorganisms interferes with the development of type I helper T cells (Th1), resulting in inadequate control of type 2 helper T cells (Th2) (6), leading to increased incidence of allergy and exacerbation of allergic disease.

Care for mild to moderate rhinitis cases include food-based approaches, such as green tea (7), and microbial stimulation based on the hygiene hypothesis. Much research has focused on non-pathogenic lactic acid bacteria and bifidobacteria (8, 9). A previous study reported that microbial stimulation, even with sterilized bacterial cells, is effective in alleviating symptoms of allergic rhinitis. The concept of paraprobiotics has been proposed to refer to such sterilized bodies or components of bacterial bodies that exhibit useful functions, as opposed to the conventional definition of probiotics, which are live microorganisms (10).

In 1953, the National Agriculture and Food Research Organization (now the National Agriculture and Food Research Organization) isolated the bacterial strain *L. paracasei* KW3110 from cheese. As a paraprobiotic, it has been shown to have various physiological functions and is suggested to be effective for patients with seasonal allergies (11). Continuous consumption of food containing *L. paracasei* KW3110 for 12 weeks reduced the severity of runny nose compared to a placebo group. Furthermore, it was confirmed that, among more than 100 types of lactic acid bacteria strains, *L. paracasei* KW3110 produced significant levels of IL-12p70, a Th1-related parameter, in the spleen cells of OVA-sensitized mice, alleviating allergic symptoms such as sneezing and nose-scratching, and reducing the production of IL-4, IL-5, and IL-13, which are involved in the exacerbation of allergies (12). It has also been reported that *L. paracasei* KW3110 acts on M2 macrophages and induces the production of large amounts of the anti-inflammatory cytokine IL-10 via dectin-2 (13, 14). Ingestion of *L. paracasei* KW3110 suppressed inflammation and photoreceptor degeneration in a mouse model of light-induced retinopathy. The mechanism of action is proposed to involve the production of IL-10 from M2 macrophages, suggesting a preventive effect against retinal degeneration. It was also reported that, in humans, *L. paracasei* KW3110 may improve eye fatigue caused by VDT load, which is accompanied by high levels of eye strain (15).

This study aimed to examine the effect of *L. paracasei* KW3110 on discomfort associated with nasal and eye allergies in subjects with mild to moderate allergic rhinitis who did not regularly use medication to relieve symptoms.

## 2 Materials and methods

### 2.1 Test and placebo tablets used in this study

*Lacticaseibacillus paracasei* KW3110 was cultured in medium, washed, pasteurized, and dried. A test tablet (total 400 mg/tablet) contained ≥ 0.7 × 10^11^ heat-killed dry *L. paracasei* KW3110 (25 mg, Kirin Holdings CO., Ltd., Tokyo, Japan), 96 mg of erythritol, 180 mg of maltose, 60 mg of *α*-cyclodextrin, 6 mg of citric acid, 1 mg of pullulan, 20 mg of cellulose, 8 mg of calcium stearate, 2 mg of yogurt powder, and 2 mg of caramel color. A placebo tablet contained 96 mg of erythritol, 205 mg of maltose, 60 mg of *α*-cyclodextrin, 6 mg of citric acid, 1 mg of pullulan, 20 mg of cellulose, 8 mg of calcium stearate, 2 mg of yogurt powder, and 2 mg of caramel color. Test and placebo tablets were prepared so that the investigators and subjects could not differentiate between them, either by appearance or smell. Subjects took two tablets, once daily.

### 2.2 Research ethics and subjects

The study adhered to the principles of the Declaration of Helsinki and the Ethical Guidelines for Medical and Health Research Involving Human Subjects. Documents such as the study protocol, the investigator’s brochure, the explanation form, and the consent form were submitted to the Human Research Ethics Committee of Kirin Holdings Company, Ltd., and the study was conducted following approval by the committee (approval number: 2023–002).

The minimum sample size was estimated on the basis of ‘runny nose’ score, which is one of the items in the Japanese Rhino-conjunctivitis Quality of Life Questionnaire (JRQLQ No.1) in the preliminary study (no. UMIN000046503). Based on an inhouse preliminary study (mean ± standard deviation for test and placebo was = 1.0 ± 0.7 and 1.4 ± 0.7, respectively), sample size calculations were performed using the Power procedure in SAS v9.4. According to these parameters, it was calculated that at least 51 subjects were needed when the power was set to 0.8 (*β* ≥ 0.8) with statistical significance *α* < 0.05 by a Mann–Whitney U test.

On the assumption that a few participants might withdraw from the study after enrollment, we determined a target number of 60 participants per group (120 in total).

**Figure 1:**
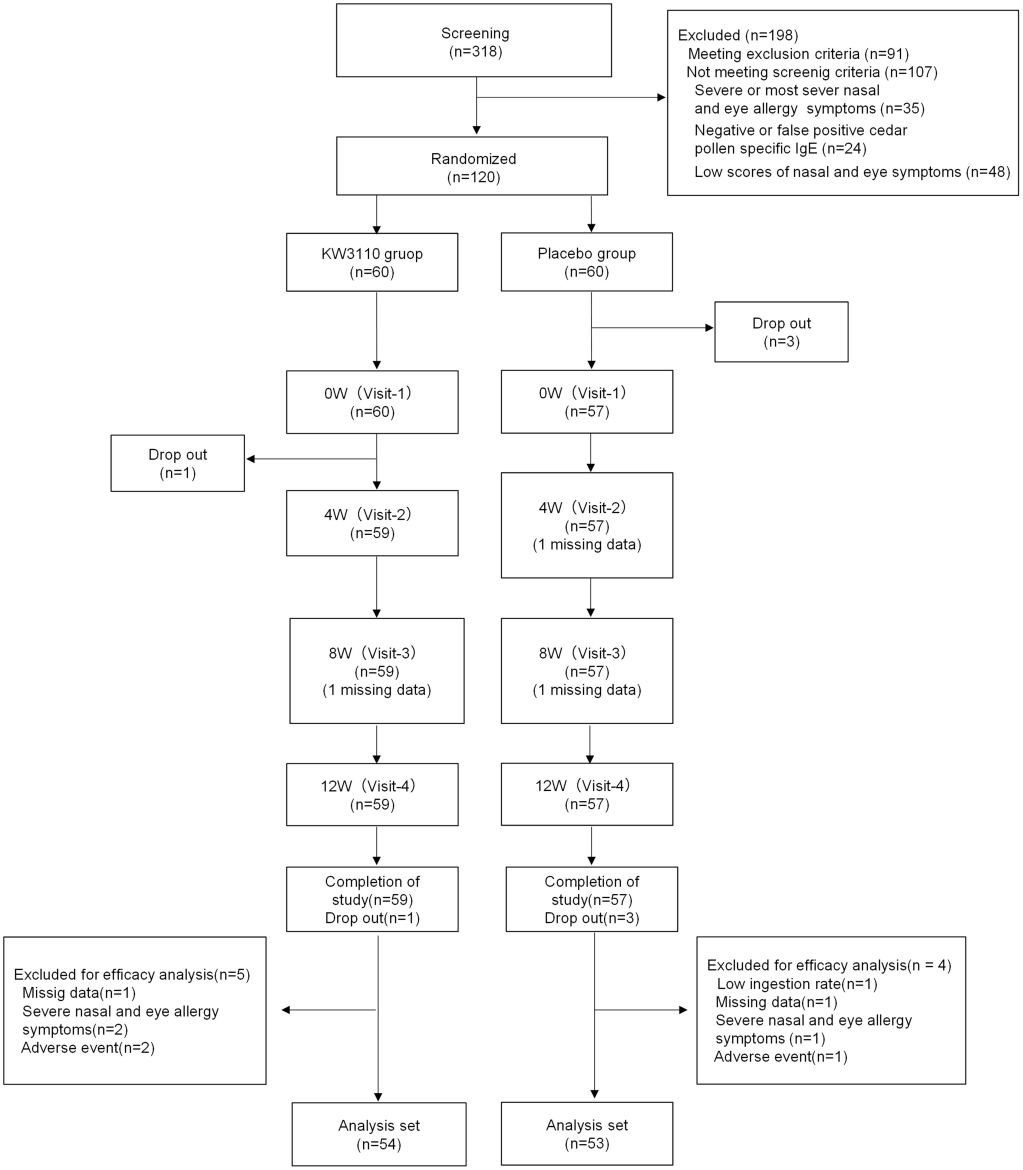
CONSORT flow diagram. “Missing data” means a subject was absent for the evaluation. W, week.

The study enrolled healthy Japanese adults (aged 20–64 years) living around Tokyo who reported nasal and eye discomfort in early spring and who did not regularly use medication to alleviate symptoms (Figure 1). Subjects were excluded from the study based on the exclusion criteria listed in Supplementary Table S1. The 120 subjects were then selected for the study after additional screening on the basis of: past symptoms being classified as mild or moderate according to the severity of allergic rhinitis symptoms; high total score for the 6 items of “nasal and eye symptoms” in the Japanese Rhino-conjunctivitis Quality of Life Questionnaire (JRQLQ No.1) for the past 2 years; class 2–6 (positive) Japanese cedar pollen specific IgE.

### 2.3 Study design

The study was registered in the University Hospital Medical Information Network (UMIN) Clinical Trials Registry (no. UMIN000053239). The study was designed as a randomized, double-blind, placebo-controlled, parallel-group trial, and was planned with TES Holdings Co., Ltd. (Tokyo, Japan), and conducted by TES Holdings Co., Ltd. and Ueno-Asagao Clinic (Tokyo, Japan).

Stratified block randomization was carried out to allocate the 120 study subjects to either the placebo group (*n* = 60) or the *L. paracasei* KW3110 group (*n* = 60). Specifically, the subjects were divided into two strata based on gender, median age, median value of nasal and eye symptoms total score in the Japanese Rhino-conjunctivitis Quality of Life Questionnaire (JRQLQ No.1) over the past 2 years, and classification of severity of allergic rhinitis symptoms by otorhinolaryngology over the past 2 years. Subjects were then randomly allocated to a group using computer-generated randomized numbers. The trial was implemented in a double-blind and placebo-controlled manner; both the data monitor and data analyst were blinded to treatment assignment until the analyses were completed.

The personnel managing the test tablets printed a mark (A or B) on the respective outer boxes enclosing the tablets and the assignment list was kept secret by an independent party until data analyses had been completed. As such, no one knew which type of tablet each subject took. Therefore, throughout this study, the subjects, site investigator, and raters involved in this study remained blinded to each subject’s assignment.

The subjects ingested two tablets of either the placebo or *L. paracasei* KW3110 per day for 12 weeks. During the study, the 120 subjects were instructed to visit the hospital five times to have an evaluation of nasal and eye symptoms by a medical doctor at weeks 0 (baseline), 4, 8, and 12 of the ingestion period.

The Japanese Rhino-conjunctivitis Quality of Life Questionnaire (JRQLQ No.1) was evaluated as a primary outcome, according to the practical guideline for the management of allergic rhinitis in Japan. The classification of severity of allergic rhinitis symptoms by otorhinolaryngology, the number of subjects who changed the classification of severity of allergic rhinitis symptoms in before and after, nasal remarks score, nasal and eye symptoms questionnaire by diary, and immunological markers were classed as secondary outcomes.

Subjects were instructed to comply with the following points during the trial: (1) avoid excessive eating and exercise; (2) have enough sleep; (3) use supplements in the same manner as they did before the trial; (4) avoid ingestion of foods containing a lot of lactic acid bacteria; and (5) avoid ingestion of medicines to reduce allergic symptoms. The study was carried out between January 2024 and April, 2024 at the laboratories of TES Holdings Co., Ltd. and Ueno-Asagao Clinic. The tablet intake period was set at 12 weeks, beginning on January 20^th^, 2024. The Japanese cedar and cypress pollen counts in the study period was measured using the Durham method by the Tokyo metropolitan government.

### 2.4 Japanese Rhino-conjunctivitis Quality of Life Questionnaire (JRQLQ No.1)

A survey of the individual subjective symptoms was conducted in accordance with the practical guidelines for the management of allergic rhinitis in Japan (2), using Japanese Rhino-conjunctivitis Quality of Life Questionnaire (JRQLQ No.1), covering “severity of nasal and eye symptoms,” “QOL-related questionnaire,” and “overall face scale” (16). Every 2 weeks (at 0, 2, 4, 6, 8, 10 and 12 weeks during the intervention period), subjective symptoms were rated using a 5-point scale: 0, no symptom; 1, mild; 2, moderate; 3, severe; 4, most severe. For the “Overall face scale,” subjects were asked to answer a face scale about their overall condition over the past 2 weeks, including their symptoms, lifestyle, and feelings, with responses ranging from “0 - cheerful” to “4 - wanting to cry.” The “Overall face scale” was calculated and scored from 0 to 4.

### 2.5 Nasal and eye symptoms questionnaire by diary

The nasal and eye symptoms questionnaire by diary was evaluated in accordance with the practical guidelines for the management of allergic rhinitis in Japan (2), covering “sneezing,” “runny nose,” “blocked nose,” “itcy eyes,” and “watery eyes.” Every day, the subjective symptoms were rated using a 5-point scale: 0, no symptom; 1, mild; 2, moderate; 3, severe; 4, most severe. Nasal and eye symptoms score was calculated from the sum of individual symptoms scores. The average value was calculated every 2 weeks.

### 2.6 Classification of severity of allergic rhinitis symptoms by otorhinolaryngology

Classification of severity of allergic rhinitis symptoms by the otorhinolaryngologist was evaluated according to the practical guidelines for the management of allergic rhinitis in Japan (2), based on a combination of paroxysmal sneezing or rhinorrhea and the severity of nasal blockage. Every 4 weeks (at 0, 4, 8, and 12 weeks during the intervention period), classification of severity of allergic rhinitis symptoms were rated by the otorhinolaryngologist using a 5-point scale: 0, no symptom; 1, mild; 2, moderate; 3, severe; 4, most severe. The improvement effect was evaluated by the otorhinolaryngologist using the following 5-point scale: 1, quite better (symptoms have disappeared); 2, better (symptoms have significantly improved); 3, improve (symptoms have improved); 4, unchanged, 5, worse.

### 2.7 Nasal remarks score

Nasal remarks score was evaluated by the otorhinolaryngologist according to the practical guidelines for the management of allergic rhinitis in Japan (2), based on swell of concha nasalis inferior mucosa and aqueous secretion. Every 4 weeks (at 0, 4, 8, and 12 weeks during the intervention period), nasal remarks score was rated by the otorhinolaryngologist using a 4-point scale: -, 1 point; +, 2 points; ++, 3 points; +++, 4 points.

### 2.8 Immunological markers

Total levels of serum IgE and cedar pollen specific IgE were measured with fluoroenzymeimmunoassay (FEIA). Peripheral blood mononuclear cells (PBMCs) were isolated from the blood using a BD Vacutainer CPT (BD Bioscience, Franklin Lakes, NJ, USA) (17). To evaluate the Th1/Th2 ratio, PBMCs were cultured in complete RPMI medium (+10 ng/mL IL-2 (Miltenyi Biotec., Bergisch Gladbach, Germany)) and supplemented with Cell Stimulation Cocktail (eBioscience, San Diego, CA, USA), before intracellular cytokines were evaluated (18). PBMCs were incubated with Zombie Green Fixable Viability Kit (Biolegend, San Diego, CA, USA), APC-Cy7 anti-human CD3 Antibody (Biolegend), Brilliant Violet 510™ anti-human CD4 Antibody (Biolegend), Alexa Fluor™ 700 anti-human CD8 (eBioscience), IFN-g Monoclonal Antibody, eFluo 450 (eBioscience), IL-4 Monoclonal Antibody, and PE (eBioscience). Data were collected using a flow cytometer (Beckman Coulter, Brea CA, USA) and analyzed using Kaluza analysis software (Beckman Coulter). Total levels of serum IgE, cedar pollen specific IgE, and Th1/Th2 ratios were assessed at 0 and 8 weeks.

### 2.9 Statistical analysis

All data values were reported as the mean ± standard error (SE). For intergroup comparisons of immunological markers, unpaired Student’s t-test was used. For intergroup comparisons of the questionnaire of the individual subjective symptoms (19–21) and evaluation by the otorhinolaryngologist, the Mann–Whitney U test was used. The number of subjects who changed their classification of severity of allergic rhinitis symptoms before and after, and the number of subjects in each group whose symptoms worsened, were unchanged, improved, improved significantly, or disappeared was calculated. Fisher’s exact test was performed if the minimum frequency was less than “5,” and Pearson’s chi-square test was performed if it was “5” or more. All statistical analyses were conducted using SAS 9.4 (SAS Institute, Cary, NC, USA) or SPSS 26 (IBM, Armonk, NY, USA). A *p*-value of <0.05 was considered to be statistically significant.

## 3 Results

### 3.1 Baseline data

No adverse event related to the test tablets was reported throughout the course of the study. The subjects were recruited as follows: first, 318 potential candidates were recruited from a sample of healthy volunteers; 91 candidates were then excluded after screening on the basis of the exclusion criteria (Supplementary Table S1). Lastly, 120 subjects from the remaining 227 were selected for the study after additional screening on the basis of: past symptoms being classified as mild or moderate according to the severity of allergic rhinitis symptoms; high total score for the 6 items of “nasal and eye symptoms” in the Japanese Rhino-conjunctivitis Quality of Life Questionnaire (JRQLQ No.1) for the past 2 years; class 2–6 (positive) Japanese cedar pollen specific IgE; and drug usage (Figure 1). Three subjects in the placebo group left the trial for health reasons just before it began. In addition, 4 subjects in the placebo group, and 5 subjects in the *L. paracasei* KW3110 group were excluded from analysis due to a low ingestion ratio (<85%), missing data for health reasons, or severe nasal and eye allergy symptoms. Thus, 107 subjects completed the trial (per-protocol set). Background data of the 107 individuals are shown in Table 1.

**Table 1:** Background data (per protocol set).

Characteristic	KW3110	Placebo	*p*-values
Number of subjects (male/female)	54 (24/30)	53 (22/31)	0.91
Age (years)	42.2 ± 1.7	44.6 ± 1.8	0.33
Severity classification of allergic rhinitis symptoms by otorhinolaryngology (past 2 years)	1.6 ± 0.1	1.6 ± 0.1	0.79
Total score for the 6 items of “nasal and eye symptoms” in the JRQLQ No.1 (past 2 years)	8.5 ± 0.3	8.8 ± 0.3	0.48

Data are expressed as the mean ± standard error (SE) with the exception of sex. The number of subjects (male/female) was evaluated using a Pearson’s chi-square test. The age of subjects was evaluated using an unpaired Student’s *t*-test. Severity classification of allergic rhinitis symptoms by otorhinolaryngology and JRQLQ No.1 were performed by the Mann–Whitney U test. There were no significant differences in any parameters between the two groups. KW3110, *L. paracasei* KW3110 group; Placebo, placebo group.

Five subjects used medications such as eye drops or nasal sprays about 1–3 times a week (3 in the *L. paracasei* KW3110 group and 2 in the placebo group), and 102 subjects did not use any medication (51 in the *L. paracasei* KW3110 group and 51 in the placebo group).

### 3.2 Environmental pollen counts

**Figure 2:**
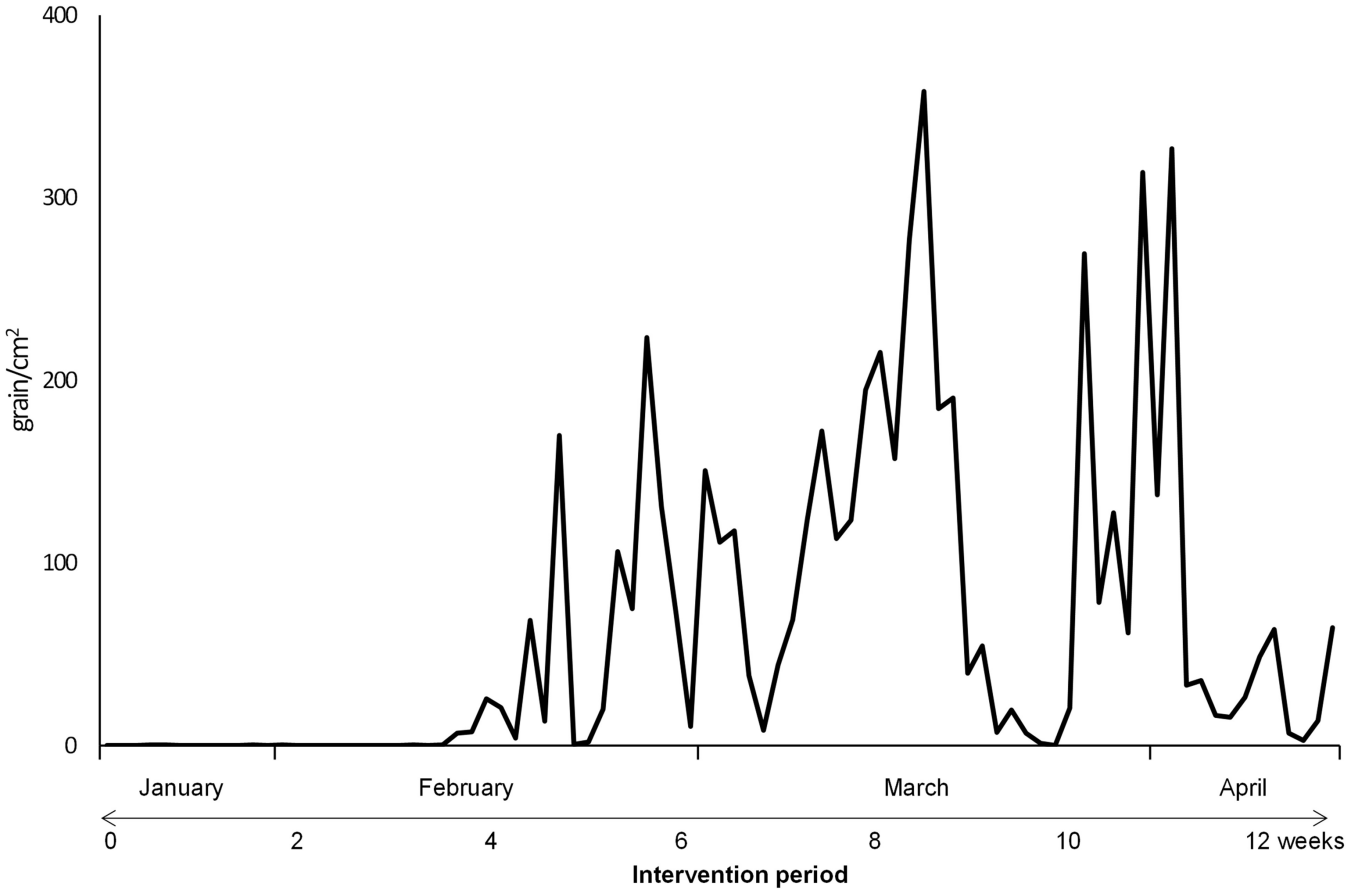
Pollen counts in Tokyo during the study period. Pollen dispersal began on February 13th, 4 weeks into the intervention period, and continued until the beginning of May. The peak of cedar pollen dispersal was recorded in mid-March, 8 weeks into the intervention period.

The Japanese cedar and cypress pollen counts in Chiyoda Ward, Tokyo, during the study period are shown in Figure 2. The cedar pollen dispersal (1 ≥ pollen grain/cm^2^) began on February 13, at 4 weeks into the intervention period, and continued until the beginning of May. The peak of cedar pollen dispersal was recorded in mid-March, at 8 weeks into the intervention period.

### 3.3 Evaluation of the nasal and eye symptoms score and the QOL-related questionnaire score of Japanese Rhino-conjunctivitis Quality of Life Questionnaire (JRQLQ No.1)

The results of the nasal and eye symptoms score and the QOL-related questionnaire score of Japanese Rhino-conjunctivitis Quality of Life Questionnaire (JRQLQ No.1) are shown in Table 2. There were no significant differences between groups before intake (0 W). In the *L. paracasei* KW3110 group, the changes in score of “nasal symptoms” and “outdoor activities” at 4 weeks were significantly lower than those in the placebo group (*p* < 0.05). Additionally, the original scores for “outdoor activities” at 2 and 4 weeks were significantly lower in the test group (*p* < 0.05, *p* < 0.01, respectively).

**Table 2:** Score of Japanese Rhino-conjunctivitis Quality of Life Questionnaire (JRQLQ No. 1).

Parameter	Group	*n*	Score							Amount of change in score					
			0 W	2 W	4 W	6 W	8 W	10 W	12 W	2 W	4 W	6 W	8 W	10 W	12 W
Runny nose	KW3110	54	0.5 ± 0.1	0.6 ± 0.1	0.6 ± 0.1	1.0 ± 0.1	1.4 ± 0.1†	1.3 ± 0.1	1.0 ± 0.1	0.1 ± 0.1	0.2 ± 0.1	0.5 ± 0.1	0.9 ± 0.1	0.8 ± 0.1	0.5 ± 0.1
	Placebo	53	0.5 ± 0.1	0.6 ± 0.1	0.8 ± 0.1	1.2 ± 0.1	1.6 ± 0.1	1.2 ± 0.1	0.9 ± 0.1	0.1 ± 0.1	0.3 ± 0.1	0.7 ± 0.1	1.1 ± 0.1	0.7 ± 0.1	0.4 ± 0.1
Sneezing	KW3110	54	0.6 ± 0.1	0.7 ± 0.1	0.9 ± 0.1	1.3 ± 0.1	1.4 ± 0.1	1.4 ± 0.1	1.1 ± 0.1	0.1 ± 0.1	0.3 ± 0.1	0.7 ± 0.1	0.8 ± 0.1	0.8 ± 0.1	0.4 ± 0.1
	Placebo	53	0.5 ± 0.1	0.7 ± 0.1	0.9 ± 0.1	1.3 ± 0.1	1.6 ± 0.1	1.3 ± 0.1	1.1 ± 0.1	0.2 ± 0.1	0.4 ± 0.1	0.8 ± 0.1	1.1 ± 0.1	0.9 ± 0.1	0.6 ± 0.1
Blocked nose	KW3110	54	0.4 ± 0.1	0.5 ± 0.1	0.6 ± 0.1	0.9 ± 0.1	1.0 ± 0.1	1.0 ± 0.1	0.7 ± 0.1	0.0 ± 0.1	0.1 ± 0.1†	0.4 ± 0.1	0.6 ± 0.1†	0.6 ± 0.1	0.3 ± 0.1
	Placebo	53	0.3 ± 0.1	0.5 ± 0.1	0.6 ± 0.1	0.9 ± 0.1	1.2 ± 0.1	1.0 ± 0.1	0.7 ± 0.1	0.1 ± 0.1	0.3 ± 0.1	0.6 ± 0.1	0.8 ± 0.1	0.7 ± 0.1	0.4 ± 0.1
Itchy nose	KW3110	54	0.2 ± 0.1	0.3 ± 0.1	0.4 ± 0.1	0.9 ± 0.1	1.0 ± 0.1	1.0 ± 0.1	0.5 ± 0.1	0.1 ± 0.1	0.1 ± 0.1	0.6 ± 0.1	0.7 ± 0.2	0.7 ± 0.2	0.3 ± 0.1
	Placebo	53	0.2 ± 0.0	0.2 ± 0.1	0.3 ± 0.1	0.9 ± 0.1	1.0 ± 0.1	0.8 ± 0.1	0.5 ± 0.1	0.1 ± 0.1	0.2 ± 0.1	0.7 ± 0.1	0.8 ± 0.1	0.7 ± 0.1	0.4 ± 0.1
Itchy eyes	KW3110	54	0.2 ± 0.1	0.4 ± 0.1	0.7 ± 0.1	1.4 ± 0.1	1.5 ± 0.1	1.3 ± 0.1	0.8 ± 0.1	0.1 ± 0.1	0.5 ± 0.1	1.2 ± 0.1	1.3 ± 0.2	1.1 ± 0.1	0.6 ± 0.1
	Placebo	53	0.3 ± 0.1	0.5 ± 0.1	0.7 ± 0.1	1.3 ± 0.1	1.6 ± 0.1	1.2 ± 0.1	0.9 ± 0.1	0.2 ± 0.1	0.4 ± 0.1	1.0 ± 0.1	1.3 ± 0.2	0.9 ± 0.1	0.6 ± 0.1
Watery eyes	KW3110	54	0.1 ± 0.0	0.1 ± 0.0	0.3 ± 0.1	0.8 ± 0.1	0.7 ± 0.1	0.7 ± 0.1	0.3 ± 0.1	0.0 ± 0.1	0.2 ± 0.1	0.6 ± 0.1	0.6 ± 0.1	0.6 ± 0.1	0.2 ± 0.1
	Placebo	53	0.1 ± 0.0	0.2 ± 0.1	0.3 ± 0.1	0.6 ± 0.1	0.7 ± 0.1	0.5 ± 0.1	0.3 ± 0.1	0.0 ± 0.0	0.2 ± 0.1	0.5 ± 0.1	0.6 ± 0.1	0.4 ± 0.1	0.2 ± 0.1
Nasal and eye symptoms	KW3110	54	2.1 ± 0.2	2.6 ± 0.3	3.5 ± 0.4	6.2 ± 0.6	7.0 ± 0.6	6.6 ± 0.7	4.4 ± 0.5	0.5 ± 0.3	1.4 ± 0.4†	4.1 ± 0.6	4.9 ± 0.6	4.5 ± 0.7	2.3 ± 0.5
	Placebo	53	1.9 ± 0.2	2.7 ± 0.3	3.6 ± 0.4	6.3 ± 0.5	7.7 ± 0.5	6.1 ± 0.4	4.4 ± 0.5	0.8 ± 0.2	1.8 ± 0.3	4.4 ± 0.5	5.8 ± 0.6	4.2 ± 0.5	2.5 ± 0.5
Nasal symptoms	KW3110	54	1.8 ± 0.2	2.1 ± 0.2	2.5 ± 0.3	4.1 ± 0.4	4.8 ± 0.4	4.6 ± 0.5	3.3 ± 0.3	0.3 ± 0.2	0.7 ± 0.3*	2.3 ± 0.4†	3.0 ± 0.4†	2.9 ± 0.5	1.5 ± 0.4
	Placebo	53	1.5 ± 0.2	2.0 ± 0.2	2.6 ± 0.2	4.3 ± 0.3	5.4 ± 0.4	4.4 ± 0.3	3.2 ± 0.3	0.6 ± 0.2	1.2 ± 0.2	2.8 ± 0.3	3.9 ± 0.4	2.9 ± 0.4	1.8 ± 0.3
Eye symptoms	KW3110	54	0.3 ± 0.1	0.5 ± 0.1	1.0 ± 0.1	2.1 ± 0.2	2.3 ± 0.2	2.0 ± 0.2	1.1 ± 0.2	0.2 ± 0.1	0.7 ± 0.2	1.8 ± 0.2	1.9 ± 0.2	1.6 ± 0.3	0.8 ± 0.2
	Placebo	53	0.4 ± 0.1	0.6 ± 0.1	1.0 ± 0.2	2.0 ± 0.2	2.3 ± 0.2	1.7 ± 0.2	1.2 ± 0.2	0.2 ± 0.1	0.6 ± 0.2	1.5 ± 0.2	1.9 ± 0.2	1.3 ± 0.2	0.7 ± 0.2
Daily life	KW3110	54	0.7 ± 0.2	0.9 ± 0.2	1.2 ± 0.3	2.6 ± 0.5	2.9 ± 0.5	2.7 ± 0.5	1.8 ± 0.4	0.2 ± 0.2†	0.5 ± 0.3	1.8 ± 0.5	2.1 ± 0.4	1.9 ± 0.5	1.1 ± 0.4
	Placebo	53	0.5 ± 0.1	0.9 ± 0.2	1.0 ± 0.2	2.5 ± 0.4	2.9 ± 0.5	2.0 ± 0.4	1.3 ± 0.3	0.4 ± 0.2	0.5 ± 0.2	2.0 ± 0.4	2.4 ± 0.5	1.5 ± 0.4	0.8 ± 0.3
Outdoor activities	KW3110	54	0.1 ± 0.1	0.1 ± 0.1*	0.2 ± 0.1**	1.0 ± 0.2	1.1 ± 0.2	1.0 ± 0.2	0.6 ± 0.1	0.0 ± 0.1	0.1 ± 0.1*	0.9 ± 0.2	1.0 ± 0.2	0.9 ± 0.2	0.5 ± 0.1
	Placebo	53	0.2 ± 0.1	0.3 ± 0.1	0.5 ± 0.1	1.3 ± 0.2	1.3 ± 0.2	1.0 ± 0.2	0.5 ± 0.1	0.1 ± 0.1	0.2 ± 0.1	1.1 ± 0.3	1.1 ± 0.2	0.8 ± 0.2	0.3 ± 0.1
Social functioning	KW3110	54	0.3 ± 0.1	0.4 ± 0.2	0.5 ± 0.2	1.0 ± 0.2	1.1 ± 0.2	1.1 ± 0.3	0.7 ± 0.2	0.1 ± 0.2	0.2 ± 0.1	0.7 ± 0.2	0.8 ± 0.2	0.8 ± 0.2	0.4 ± 0.2
	Placebo	53	0.3 ± 0.1	0.3 ± 0.1	0.4 ± 0.1	1.1 ± 0.2	1.2 ± 0.2	1.1 ± 0.2	0.6 ± 0.2	0.0 ± 0.1	0.2 ± 0.1	0.9 ± 0.2	0.9 ± 0.2	0.9 ± 0.2	0.3 ± 0.2
Sleep disurbance	KW3110	54	0.1 ± 0.1	0.1 ± 0.0	0.1 ± 0.1	0.5 ± 0.1	0.5 ± 0.1	0.4 ± 0.1	0.3 ± 0.1	0.0 ± 0.0	0.0 ± 0.1	0.3 ± 0.1	0.4 ± 0.1	0.3 ± 0.1	0.1 ± 0.1
	Placebo	53	0.2 ± 0.1	0.2 ± 0.1	0.2 ± 0.1	0.4 ± 0.1	0.5 ± 0.1	0.3 ± 0.1	0.2 ± 0.1	0.0 ± 0.1	0.0 ± 0.1	0.2 ± 0.1	0.3 ± 0.1	0.1 ± 0.1	0.0 ± 0.1
Physical problems	KW3110	54	0.4 ± 0.1	0.4 ± 0.1	0.5 ± 0.1	1.1 ± 0.2	1.1 ± 0.2	0.9 ± 0.2	0.6 ± 0.2	0.1 ± 0.1	0.1 ± 0.1	0.7 ± 0.2	0.7 ± 0.2	0.5 ± 0.2	0.3 ± 0.2
	Placebo	53	0.2 ± 0.1	0.4 ± 0.1	0.4 ± 0.1	1.0 ± 0.2	1.2 ± 0.2	1.0 ± 0.2	0.7 ± 0.1	0.2 ± 0.1	0.2 ± 0.1	0.7 ± 0.2	1.0 ± 0.2	0.7 ± 0.2	0.5 ± 0.1
Emotional function	KW3110	54	0.4 ± 0.2	0.4 ± 0.1	0.7 ± 0.3	1.8 ± 0.4	1.7 ± 0.4	1.7 ± 0.4	1.1 ± 0.3	−0.1 ± 0.1†	0.3 ± 0.2	1.3 ± 0.4	1.3 ± 0.4	1.3 ± 0.4	0.7 ± 0.3
	Placebo	53	0.2 ± 0.1	0.5 ± 0.1	0.5 ± 0.2	1.6 ± 0.4	1.6 ± 0.4	1.3 ± 0.3	0.8 ± 0.2	0.2 ± 0.1	0.3 ± 0.2	1.4 ± 0.3	1.4 ± 0.4	1.0 ± 0.3	0.6 ± 0.2
QOL-related questions	KW3110	54	2.1 ± 0.6	2.4 ± 0.7	3.3 ± 1.0	7.8 ± 1.6	8.4 ± 1.5	7.7 ± 1.6	5.2 ± 1.1	0.4 ± 0.6†	1.2 ± 0.9	5.8 ± 1.5†	6.3 ± 1.4	5.7 ± 1.5	3.1 ± 1.1
	Placebo	53	1.7 ± 0.4	2.6 ± 0.6	3.0 ± 0.7	7.9 ± 1.3	8.8 ± 1.4	6.7 ± 1.2	4.0 ± 0.9	0.9 ± 0.4	1.4 ± 0.6	6.2 ± 1.3	7.1 ± 1.4	5.0 ± 1.2	2.3 ± 0.9
Overall face scale	KW3110	54	1.1 ± 0.1	1.1 ± 0.1	1.2 ± 0.1	1.6 ± 0.1	1.8 ± 0.1	1.6 ± 0.1	1.3 ± 0.1	0.0 ± 0.1	0.2 ± 0.1	0.5 ± 0.1	0.8 ± 0.1	0.6 ± 0.1	0.2 ± 0.1
	Placebo	53	1.2 ± 0.1	1.2 ± 0.1	1.4 ± 0.1	1.8 ± 0.1	2.0 ± 0.1	1.7 ± 0.1	1.4 ± 0.1	0.0 ± 0.1	0.1 ± 0.1	0.5 ± 0.1	0.8 ± 0.1	0.5 ± 0.1	0.1 ± 0.1

Mean ± standard error. †*p* < 0.10, *p < 0.05, **p < 0.01 (Mann–Whitney U test between the two groups). “Amount of change in score” is the score of difference from 0 W.

### 3.4 Evaluation of the nasal and eye symptoms questionnaire by diary

Nasal and eye symptom scores collected by diary questionnaire are shown in Supplementary Table S2. There were no significant differences between groups before intake (0 W). In the *L. paracasei* KW3110 group, the original score and the change in the score of “runny nose” at 8 weeks were significantly lower than in the placebo group (*p* < 0.05).

### 3.5 Severity classification of allergic rhinitis symptoms by otorhinolaryngology

The results of the classification of the severity of allergic rhinitis symptoms are shown in Table 3. There were no significant differences between groups before intake (0 W). In the *L. paracasei* KW3110 group, the changes in the “paroxysmal sneezing or rhinorrhea score” and “classification of severity of allergic rhinitis symptoms score” at 4 weeks were significantly lower than those in the placebo group (*p* < 0.05).

**Table 3:** Classification of severity of allergic rhinitis symptoms by otorhinolaryngology.

Parameter	Group	*n*	Score				Amount of change in score		
			0 W	4 W	8 W	12 W	4 W	8 W	12 W
Paroxysmal sneezing or rhinorrhea	KW3110	54	0.9 ± 0.1	1.0 ± 0.1†	1.4 ± 0.1	0.9 ± 0.1	0.1 ± 0.1*	0.6 ± 0.1	0.1 ± 0.1
	Placebo	53	0.8 ± 0.1	1.2 ± 0.1	1.7 ± 0.1	0.9 ± 0.1	0.4 ± 0.1	0.8 ± 0.1	0.1 ± 0.1
Nasal blockage	KW3110	54	0.5 ± 0.1	0.5 ± 0.1	1.0 ± 0.1	0.5 ± 0.1	0.1 ± 0.1	0.5 ± 0.1	0.0 ± 0.1
	Placebo	53	0.5 ± 0.1	0.6 ± 0.1	1.1 ± 0.1	0.5 ± 0.1	0.2 ± 0.1	0.6 ± 0.1	0.0 ± 0.1
Classification of severity of allergic rhinitis symptoms	KW3110	54	0.9 ± 0.1	1.0 ± 0.1†	1.5 ± 0.1	0.9 ± 0.1	0.1 ± 0.1*	0.6 ± 0.1	0.1 ± 0.1
	Placebo	53	0.9 ± 0.1	1.3 ± 0.1	1.7 ± 0.1	1.1 ± 0.1	0.4 ± 0.1	0.8 ± 0.1	0.2 ± 0.1

Mean ± standard error. †*p* < 0.10, *p < 0.05 (Mann–Whitney U test between the two groups). “Amount of change in score” is the score of difference from 0 W.

The number of subjects that changed the severity classification of allergic rhinitis symptoms between before and after the trial are shown in Table 4. At 4 weeks, the *L. paracasei* KW3110 group had a significantly smaller proportion of those classified as “worse” or “unchanged” compared to the placebo group (*p* < 0.01). No significant difference was observed between the two groups at 8 and 12 weeks (Supplementary Tables S3, S4).

**Table 4:** Number of subjects changed the classification of severity of allergic rhinitis symptoms before and after 4 weeks.

Evaluation	Change in number from 0-4 W				Fisher’s exact test
	KW3110 (*n* = 54)		Placebo (*n* = 52)		
Worse	10*	18.5%	22	42.3%	*p* = 0.0084
Unchanged	41**	75.9%	25	48.1%	
Improved	1	1.9%	3	5.8%	
Improved Significantly	0	0.0%	1	1.9%	
Disappeared	2	3.7%	1	1.9%	
Total	54	100.0%	52	100.0%	

Between-group comparisons of the number of people corresponding to each effect assessment were performed using Fisher’s exact test. To assess each effect, subjects were divided into two strata: applicable and non-applicable, and between-group comparisons of numbers were performed using Pearson’s chi-square test. *p < 0.05, **p < 0.01. 1 subject in the placebo group was excluded from analysis due to missing data for health reason.

### 3.6 Evaluation of nasal remarks score

Nasal remarks scored by the otolaryngologist are shown in Supplementary Table S5. There were no significant differences between groups before intake (0 W). In the *L. paracasei* KW3110 group, the original score of “swell of concha nasalis inferior mucosa” at 4 weeks was significantly lower than that in the placebo group (*p* < 0.05).

### 3.7 Immunological markers

No significant difference was observed between the two groups (Supplementary Table S6).

## 4 Discussion

We investigated the effects of consuming tablets containing *L. paracasei* KW3110 on nasal and eye discomfort in healthy subjects, aged 20 to 64 years, with mild to moderate allergic rhinitis for 12 consecutive weeks. Subjects reported that they experienced nasal and eye discomfort in early spring but did not regularly use medication to alleviate symptoms. Our results showed there were significant improvements in the *L. paracasei* KW3110 group when compared with the placebo group in the score of: “nasal symptoms” and “outdoor activities” of the JRQLQ No.1 at 4 weeks; “runny nose” in the nasal and eye symptoms questionnaire by diary at 8 weeks; the score of “paroxysmal sneezing or rhinorrhea”; and “classification of severity of allergic rhinitis symptoms” by the otorhinolaryngologist at 4 weeks. There was also a significant decrease in the number of subjects who experienced “worse” in the efficacy assessment, suggesting that the continued intake of tablets containing *L. paracasei* KW3110 contributed to the prevention of nasal discomfort.

It has been reported that during the development of type I allergies, such as allergic rhinitis, cell wall components of lactic acid bacteria act on antigen-presenting cells and induce cytokines such as IL-12 (22, 23). For example, cell wall-associated heat shock proteins GroES and GroEL of *L. acidophilus* L-92 are reported to be involved in the production of cytokines such as IL-12 (22). While it has been shown that *Enterococcus faecalis* strain EC-12 is phagocytosed by antigen-presenting cells when mannose in the cell wall is recognized by mannose receptors, resulting in the production of IL-12 (23). It is suggested that IL-12 strongly induces Th1 cells, which weaken the activity of Th2 cells, thereby reducing a series of allergic reactions caused by Th2 cells (24–27). It is suggested that nasal and eye symptoms are alleviated by a factor which induces the production of IL-12 from antigen-presenting cells and suppresses Th2. It has been reported that orally ingested *L. paracasei* KW3110 reaches the intestinal tract, and is taken up by CD11b-positive cells in Peyer’s patch, resulting in the production of IL-12 (27). Previous *in vivo* studies have confirmed that *L. paracasei* KW3110 improves IL-12 production and alleviates allergic symptoms (12). In addition, the results of this study showed that the change in Th1/Th2 ratio was higher in the *L. paracasei* KW3110 group than in the placebo group, although the difference was not significant (Supplementary Table S6). Therefore, we suggest that the alleviating effects of *L. paracasei* KW3110 on rhinitis symptoms observed in this study were due to antigen-presenting cells such as dendritic cells recognizing the bacteria, which in turn produce cytokines that regulate the function of Th1/Th2 immune cells. Many studies have been conducted on the mechanism by which lactic acid bacteria alleviate allergic symptoms. Since sterilized, dead *L. paracasei* KW3110 bacteria alleviated rhinitis symptoms in this study, it is highly likely that it is the cell wall components of *L. paracasei* KW3110 that are the trigger; however, further research is needed to clarify this.

Previous studies have shown that *L. paracasei* KW3110 also acts on M2 macrophages and induces the production of large amounts of IL-10, an anti-inflammatory cytokine (13). Recognition of *α*-mannose on the cell wall triggers IL-10 production and phagocytosis by dectin-2 in macrophages (15). IL-10, an anti-inflammatory cytokine, is thought to suppress allergic reactions and may also be involved in the alleviation of allergic rhinitis (28). In addition, it has been reported that the production of IL-10 by M2 macrophages suppresses airway allergy (29). Although IL-10 production was not evaluated in this study, it is possible that *L. paracasei* KW3110 not only induced Th1, but also improved the balance of mutual control between immune cells by increasing IL-10 production, thereby controlling the activity of excessive Th2 cells, and improving allergic symptoms.

Morita et al. reported IL-10 production after ingestion of *L. paracasei* KW3110 suppressed inflammation in the eyes, thereby reducing eye fatigue (13, 15). We observed a reduction in nasal symptoms in the *L. paracasei* KW3110 group, but no effect on ocular symptoms was confirmed. In several studies using other lactic acid bacteria and acetic acid bacteria, the effect was only comfirmed in the nose, so it was thought that it would be difficult to confirm the effect in the eyes (30, 31).

Consuming functional foods that regulate the Th1/Th2 balance or have a suppressive effect on Th2 before the pollen season may help to suppress the excess (or imbalance) of Th2 when pollen is released, thereby alleviating allergic symptoms, as in our study. The effects obtained in this study are appropriate for a functional food that is expected to play a preventive role, as opposed to a drug intended for treatment. However, in this study, almost no effect of *L. paracasei* KW3110 was observed from week 10 onwards, when the amount of pollen released in the air increased. The reason for this may be that the amount of pollen antigens was excessive, and the amount of *L. paracasei* KW3110 consumed in this study was not enough to suppress the increase in Th2. This may have resulted in an increase in mast cells binding pollen-specific IgE (establishment of sensitization), and therefore the subsequent mast cell response to the pollen antigens could not be suppressed.

Subjects who had used nasal or eye drops 1–3 times a week before this study were included because it was believed they would experience more pronounced allergic symptoms and the tablets would produce a greater effect on their allergic symptoms. However, *L. paracasei* KW3110 produced a more pronounced effect in subjects who had not used medication. In the JRQLQ No.1, significant improvement was confirmed in the *L. paracasei* KW3110 group in “nasal symptoms (amount of change)” category group compared to the placebo group at 4 weeks, and when pollen dispersion had increased, at 6 weeks (Supplementary Table S8). Additionally, significant improvement was confirmed in the *L. paracasei* KW3110 group compared to the placebo group in the “runny nose (original scores)” category, at 8 weeks (Supplementary Table S8). Furthermore, in the “runny nose” category in the nasal and eye symptoms questionnaire by diary, significant improvement was confirmed compared to the placebo group at both 8 and 10 weeks (Supplementary Table S10). Additionally, in the “blocked nose” category, significant improvement was confirmed in the test group compared to the placebo group at 8 weeks (Supplementary Table S10). It is suggested that medication use caused changes in rhinitis symptoms, making it difficult to evaluate the accuracy effects of the tablets due to the effects of the medication. That is, *L. paracasei* KW3110 showed a greater effect in alleviating allergy symptoms in subjects who did not use medication because the effects of *L. paracasei* KW3110 alone could be evaluated more accurately.

In this study, the Th1/Th2 ratio was analyzed using PBMCs collected from the subjects’ blood, but no significant change was observed. Therefore, it is difficult to determine the anti-allergic mechanism of *L. paracasei* KW3110 in this study. Considering the results of previous *in vitro* and *in vivo* studies, it was speculated that IL-12 and IL-10 were involved (12, 13), but they were not detected in this analysis. In future studies, it will be necessary to devise measurement methods so that blood indicators can also be confirmed. Specifically, by carrying out quantitative analysis of Th1/Th2 during subject selection, and targeting subjects with relatively consistent Th1/Th2 status, it may be possible to more accurately evaluate the effect of *L. paracasei* KW3110 on immune cells. Alternatively, it may be possible to confirm the mechanism of action in reducing nasal discomfort by evaluating the amount of IL-12 and IL-10 produced by antigen-presenting cells that *L. paracasei* KW3110 directly acts on.

There were several limitations in this study. First, this study confirmed a significant improvement in rhinitis symptoms in subjects with relatively mild to moderate symptoms. However, because the study did not target subjects with severe nasal and eye symptoms, the effects on patients with severe nasal symptoms are unclear from the results of this study. Second, as mentioned above, we observed a reduction in nasal symptoms, but no improvement in eye symptoms was confirmed. Third, as mentioned above, the Th1/Th2 ratio was analyzed, but no significant changes were observed, so it is difficult to determine the mechanism of the anti-allergic effect of *L. paracasei* KW3110 in this study. Further research into these issues is needed.

## 5 Conclusion

Intake of *L. paracasei* KW3110 was effective in alleviating allergic rhinitis symptoms and related discomfort in healthy subjects who do not regularly use medication, especially at the beginning of the pollen dispersal period. In the future, the mechanisms that contribute to the relief of allergic rhinitis need to be clarified in humans. The findings from the present and future studies might be useful for the development and proposal of preventive solutions for people suffering from allergic rhinitis, including hay fever.

## Data Availability

The original contributions presented in the study are included in the article/Supplementary material, further inquiries can be directed to the corresponding author.
