# Supplementary material for: Effect of heat-killed Lacticaseibacillus paracasei KW3110 on mild to moderate seasonal allergic rhinitis symptoms in Japanese adults: a randomized, double-blind, placebo-controlled parallel-group study
Source: Front Nutr. 2025 Jun 25;12:1568329. doi: 10.3389/fnut.2025.1568329 (PMC12239017; doi:10.3389/fnut.2025.1568329)
Supplement: Supplementary file 1 [file Table_1.docx]

Supplementary Material

# Supplementary Tables

**Table S1.** Exclusion criteria in the study.

| 1) | Subjects who have some diseases with drug therapy. |
| --- | --- |
| 2) | Subjects who use oral antiallergic drugs and use regularly antiallergic eye and nasal drops, or will use during the test period. |
| 3) | Subjects who have history of intranasal laser therapy or sublingual immunotherapy to reduce allergic symptoms. |
| 4) | Subjects who are a patient of or have a history of asthma. |
| 5) | Subjects who are a patient of or have a history of atopic dermatitis or chronic urticaria. |
| 6) | Subjects who are a patient or have a history of mental disease, psychiatric disease, high blood pressure, diabetes, and hyperlipidemia. |
| 7) | Subjects who consecutively receive drugs for treatment of disease in the last 1 month. |
| 8) | Subjects who could have severe allergy to foods or drugs. |
| 9) | Subjects who have severe disease histories in liver, kidney, heart, lung, blood or the other tissues. |
| 10) | Subjects who are a patient or have a history of endocrine disease. |
| 11) | Subjects whose BMI is over 30 kg/m^2^. |
| 12) | Subjects who have donated over 200 mL of blood in the last 1 month or 400 mL of blood in the last 3 months. |
| 13) | Subjects who cannot stop to ingest yogurt or intestinal drugs or supplements with lactic acid bacteria or bifidobacterium during the test period. |
| 14) | Subjects who had a habit to ingest functional foods with bifidobacterium or health-promoting foods, health foods, or supplements containing similar composition with a test food or will ingest those foods during the test period. |
| 15) | Subjects who generally intake alcohol drinks over 60 g/day as the amount of pure alcohol. |
| 16) | Subjects who could change their location and their life style during the study, e.g. business trip or traveling for a long time. |
| 17) | Subjects who are pregnant, breastfeeding, or planning to be pregnant. |
| 18) | Subjects who participated in other clinical trials in the last 3 months. |
| 19) | Subjects who and whose family work for a company manufacturing or selling healthy foods, functional foods. |
| 20) | Subjects who refuse to disclose their biological sex. |
| 21) | Subjects who are judged unsuitable for this study by the investigator for other reasons. |

**Table S2.** Scores of Nasal and Eye Symptoms Questionnaire by diary.

|  |  |  | Score | | | | | | | | | | | | | | | | | | Amount of change in score | | | | | | | | | | | | | | |
| --- | --- | --- | --- | --- | --- | --- | --- | --- | --- | --- | --- | --- | --- | --- | --- | --- | --- | --- | --- | --- | --- | --- | --- | --- | --- | --- | --- | --- | --- | --- | --- | --- | --- | --- | --- |
| Parameter | Group | n | 2W | | | 4W | | | 6W | | | 8W | | | 10W | | | 12W | | | 4W | | | 6W | | | 8W | | | 10W | | | 12W | | |
| Sneezing | KW3110 | 54 | 0.53 | ± | 0.05 | 0.68 | ± | 0.07 | 0.96 | ± | 0.08 | 1.12 | ± | 0.09 | 1.06 | ± | 0.09 | 0.91 | ± | 0.10 | 0.15 | ± | 0.05 | 0.43 | ± | 0.08 | 0.59 | ± | 0.09 | 0.52 | ± | 0.09 | 0.37 | ± | 0.09 |
|  | Placebo | 53 | 0.54 | ± | 0.07 | 0.65 | ± | 0.08 | 0.97 | ± | 0.07 | 1.25 | ± | 0.09 | 1.20 | ± | 0.10 | 0.99 | ± | 0.09 | 0.11 | ± | 0.04 | 0.43 | ± | 0.05 | 0.71 | ± | 0.09 | 0.66 | ± | 0.09 | 0.46 | ± | 0.08 |
| Runny nose | KW3110 | 54 | 0.68 | ± | 0.07 | 0.73 | ± | 0.07 | 0.93 | ± | 0.09 | 1.10 | ± | 0.10* | 1.08 | ± | 0.10† | 0.88 | ± | 0.09 | 0.05 | ± | 0.06 | 0.24 | ± | 0.08 | 0.41 | ± | 0.10* | 0.39 | ± | 0.10† | 0.19 | ± | 0.10† |
|  | Placebo | 53 | 0.67 | ± | 0.08 | 0.74 | ± | 0.08 | 1.01 | ± | 0.09 | 1.41 | ± | 0.11 | 1.33 | ± | 0.11 | 1.05 | ± | 0.10 | 0.08 | ± | 0.05 | 0.34 | ± | 0.07 | 0.74 | ± | 0.10 | 0.66 | ± | 0.10 | 0.39 | ± | 0.09 |
| Blocked nose | KW3110 | 54 | 0.32 | ± | 0.06 | 0.43 | ± | 0.07 | 0.54 | ± | 0.08 | 0.70 | ± | 0.09 | 0.67 | ± | 0.09 | 0.50 | ± | 0.08 | 0.11 | ± | 0.05 | 0.22 | ± | 0.08 | 0.38 | ± | 0.10† | 0.35 | ± | 0.10 | 0.19 | ± | 0.09 |
|  | Placebo | 53 | 0.26 | ± | 0.06 | 0.36 | ± | 0.07 | 0.56 | ± | 0.07 | 0.88 | ± | 0.10 | 0.76 | ± | 0.10 | 0.57 | ± | 0.09 | 0.10 | ± | 0.03 | 0.30 | ± | 0.06 | 0.62 | ± | 0.08 | 0.49 | ± | 0.10 | 0.31 | ± | 0.09 |
| Itcy eyes | KW3110 | 54 | 0.21 | ± | 0.06 | 0.39 | ± | 0.08 | 1.00 | ± | 0.12 | 1.18 | ± | 0.12 | 1.03 | ± | 0.10 | 0.66 | ± | 0.10 | 0.19 | ± | 0.05 | 0.79 | ± | 0.12 | 0.98 | ± | 0.13 | 0.83 | ± | 0.11 | 0.45 | ± | 0.11 |
|  | Placebo | 53 | 0.25 | ± | 0.06 | 0.37 | ± | 0.08 | 0.84 | ± | 0.08 | 1.29 | ± | 0.11 | 1.01 | ± | 0.11 | 0.73 | ± | 0.10 | 0.12 | ± | 0.04 | 0.60 | ± | 0.07 | 1.05 | ± | 0.10 | 0.77 | ± | 0.12 | 0.49 | ± | 0.10 |
| Watery eyes | KW3110 | 54 | 0.09 | ± | 0.03 | 0.16 | ± | 0.05 | 0.43 | ± | 0.08 | 0.53 | ± | 0.09 | 0.48 | ± | 0.09 | 0.31 | ± | 0.08 | 0.07 | ± | 0.03 | 0.34 | ± | 0.08 | 0.44 | ± | 0.08 | 0.39 | ± | 0.09 | 0.23 | ± | 0.07 |
|  | Placebo | 53 | 0.12 | ± | 0.04 | 0.17 | ± | 0.05 | 0.39 | ± | 0.07 | 0.52 | ± | 0.08 | 0.40 | ± | 0.09 | 0.27 | ± | 0.07 | 0.06 | ± | 0.03 | 0.28 | ± | 0.04 | 0.40 | ± | 0.07 | 0.28 | ± | 0.08 | 0.15 | ± | 0.06 |

Mean ± standard error.

†p < 0.10, *p < 0.05 (the Mann–Whitney U test between the two groups).

"Amount of change in score" is the score of difference from 0W.

**Table S3.** Number of subjects changed the classification of severity of allergic rhinitis symptoms before and after 8 weeks.

| Evaluation | Change in number from 0-8W | | | | Fisher's exact test | | | |
| --- | --- | --- | --- | --- | --- | --- | --- | --- |
|  | KW3110 (n=54) | | Placebo (n=53) | |  |  |  |  |
| worse | 28 | 51.9% | 34 | 64.2% | *P* | = | 0.1069 |  |
| unchanged | 25† | 46.3% | 15 | 28.3% |  |  |  |  |
| improved | 0 | 0.0% | 2 | 3.8% |  |  |  |  |
| improved significantly | 0 | 0.0% | 1 | 1.9% |  |  |  |  |
| disappeared | 1 | 1.9% | 1 | 1.9% |  |  |  |  |
| total | 54 | 100.0% | 53 | 100.0% |  |  |  |  |

Between-group comparisons of the number of people corresponding to each effect assessment were performed using Fisher's exact test.

To assess each effect, participants were divided into two strata: applicable and non-applicable, and between-group comparisons of numbers were performed using Pearson's chi-square test. †p < 0.10

**Table S4.** Number of subjects changed the classification of severity of allergic rhinitis symptoms before and after 12 weeks.

| Evaluation | Change in number from 0-12W | | | | Fisher's exact test | | | |
| --- | --- | --- | --- | --- | --- | --- | --- | --- |
|  | KW3110 (n=54) | | Placebo (n=53) | |  |  |  |  |
| worse | 14 | 25.9% | 16 | 30.2% | *P* | = | 0.7872 |  |
| unchanged | 30 | 55.6% | 31 | 58.5% |  |  |  |  |
| improved | 4 | 7.4% | 3 | 5.7% |  |  |  |  |
| improved significantly | 0 | 0.0% | 0 | 0.0% |  |  |  |  |
| disappeared | 6 | 11.1% | 3 | 5.7% |  |  |  |  |
| total | 54 | 100.0% | 53 | 100.0% |  |  |  |  |

Between-group comparisons of the number of people corresponding to each effect assessment were performed using Fisher's exact test.

To assess each effect, subjects were divided into two strata: applicable and non-applicable, and between-group comparisons of numbers were performed using Pearson's chi-square test.

**Table S5.** Nasal remarks score.

|  |  |  | Score | | | | | | | | | | | | Amount of change in score | | | | | | | | |
| --- | --- | --- | --- | --- | --- | --- | --- | --- | --- | --- | --- | --- | --- | --- | --- | --- | --- | --- | --- | --- | --- | --- | --- |
| Parameter | Group | n | 0W | | | 4W | | | 8W | | | 12W | | | 4W | | | 8W | | | 12W | | |
| Swell of concha nasalis inferior mucosa | KW3110 | 54 | 1.3 | ± | 0.1† | 1.3 | ± | 0.1* | 1.6 | ± | 0.1 | 1.2 | ± | 0.1† | 0.0 | ± | 0.1 | 0.3 | ± | 0.1 | -0.1 | ± | 0.1 |
|  | Placebo | 53 | 1.5 | ± | 0.1 | 1.7 | ± | 0.1 | 1.8 | ± | 0.1 | 1.4 | ± | 0.1 | 0.2 | ± | 0.1 | 0.3 | ± | 0.1 | -0.2 | ± | 0.1 |
| Aqueous secretion | KW3110 | 54 | 1.7 | ± | 0.1 | 1.9 | ± | 0.1 | 2.1 | ± | 0.1 | 1.7 | ± | 0.1 | 0.2 | ± | 0.1 | 0.4 | ± | 0.1 | 0.0 | ± | 0.1 |
|  | Placebo | 53 | 1.8 | ± | 0.1 | 1.9 | ± | 0.1 | 2.3 | ± | 0.1 | 1.8 | ± | 0.1 | 0.1 | ± | 0.1 | 0.5 | ± | 0.1 | 0.0 | ± | 0.1 |

Mean ± standard error.

†p < 0.10, *p < 0.05 (the Mann–Whitney U test between the two groups).

"Amount of change in score" is the score of difference from 0W.

**Table S6.** Immunological markers.

|  |  |  |  | Score | | | | | | Amount of change in score | | |
| --- | --- | --- | --- | --- | --- | --- | --- | --- | --- | --- | --- | --- |
| Parameter | Unit | Group | n | 0W | | | 8W | | | 8W | | |
| Total IgE | IU/mL | KW3110 | 54 | 198.3 | ± | 37.4 | 162.6 | ± | 290.0 | -35.7 | ± | 12.9 |
|  |  | Placebo | 53 | 298.7 | ± | 93.3 | 250.5 | ± | 70.1 | -48.2 | ± | 26.0 |
| Cedar pollen IgE | － | KW3110 | 54 | 14.52 | ± | 2.27 | 13.89 | ± | 2.19 | -0.63 | ± | 0.50 |
|  |  | Placebo | 53 | 15.32 | ± | 2.66 | 15.34 | ± | 2.41 | 0.02 | ± | 0.64 |
| Th1 (CD4+IFN-γ+) | % | KW3110 | 54 | 15.29 | ± | 0.95 | 18.70 | ± | 1.03 | 3.42 | ± | 0.68 |
|  |  | Placebo | 53 | 16.59 | ± | 0.88 | 18.95 | ± | 0.89 | 2.37 | ± | 0.63 |
| Th2 (CD4+IL-4+) | % | KW3110 | 54 | 5.651 | ± | 0.271 | 6.494 | ± | 0.281 | 0.842 | ± | 0.179 |
|  |  | Placebo | 53 | 6.333 | ± | 0.370 | 6.860 | ± | 0.410 | 0.528 | ± | 0.193 |
| Th1/Th2 | － | KW3110 | 54 | 2.956 | ± | 0.210 | 3.239 | ± | 0.258 | 0.284 | ± | 0.153 |
|  |  | Placebo | 53 | 3.053 | ± | 0.246 | 3.252 | ± | 0.256 | 0.198 | ± | 0.111 |

Mean ± standard error. "Amount of change in score" is the score of difference from 0W.

**Table S7.** Background data (subjects who did not use medication).

| Characteristic | KW3110 | | | Placebo | | | p-value | |
| --- | --- | --- | --- | --- | --- | --- | --- | --- |
| Number of subjects (male/female) | 51 (24/27) | | | 51(21/30) | | | *P*= | 0.69 |
| Age (years) | 42.2 | ± | 1.7 | 44.2 | ± | 1.8 | *P*= | 0.42 |
| Severity classification of allergic rhinitis symptoms  by otorhinolaryngology（past 2 years） | 1.6 | ± | 0.1 | 1.6 | ± | 0.1 | *P*= | 0.68 |
| Total score for the 6 items of "nasal and eye symptoms"  in the JRQLQ No.1（past 2 years） | 8.5 | ± | 0.4 | 8.7 | ± | 0.3 | *P*= | 0.59 |

Data are expressed as the mean ± standard error with the exception of sex. The number of subjects (male/female) was evaluated using a Pearson's chi-square test. The age of subjects was evaluated using an unpaired Student’s t-test.

Severity classification of allergic rhinitis symptoms by otorhinolaryngology and JRQLQ No.1 were performed by the Mann–Whitney U test.

There was no significant difference in any parameters between the two groups.

KW3110 = *L. paracasei* KW3110 group. placebo = placebo group.

**Table S8.** Scores of Japanese Rhinoconjunctivitis Quality of Life Questionnaire (JRQLQ No.1) (subjects who did not use medication).

|  |  |  | Score | | | | | | | | | | | | | | | | | | | | | | Amount of change in score | | | | | | | | | | | | | | | | | | | | | | |
| --- | --- | --- | --- | --- | --- | --- | --- | --- | --- | --- | --- | --- | --- | --- | --- | --- | --- | --- | --- | --- | --- | --- | --- | --- | --- | --- | --- | --- | --- | --- | --- | --- | --- | --- | --- | --- | --- | --- | --- | --- | --- | --- | --- | --- | --- | --- | --- |
| Parameter | Group | n | 0W | | | 2W | | | 4W | | | 6W | | | 8W | | | 10W | | | 12W | | | | 2W | | | | 4W | | | | 6W | | | | 8W | | | | 10W | | | | 12W | | |
| Runny nose | KW3110 | 51 | 0.5 | ± | 0.1 | 0.6 | ± | 0.1 | 0.6 | ± | 0.1 | 1.0 | ± | 0.1† | 1.3 | ± | 0.1* | 1.2 | ± | 0.1 | 0.9 | ± | 0.1 | 0.1 | | ± | 0.1 | 0.2 | | ± | 0.1 | 0.5 | | ± | 0.1 | 0.8 | | ± | 0.1 | 0.8 | | ± | 0.1 | 0.5 | | ± | 0.1 |
|  | Placebo | 51 | 0.5 | ± | 0.1 | 0.6 | ± | 0.1 | 0.8 | ± | 0.1 | 1.2 | ± | 0.1 | 1.6 | ± | 0.1 | 1.2 | ± | 0.1 | 1.0 | ± | 0.1 | 0.1 | | ± | 0.1 | 0.3 | | ± | 0.1 | 0.7 | | ± | 0.1 | 1.1 | | ± | 0.1 | 0.6 | | ± | 0.1 | 0.4 | | ± | 0.1 |
| Sneezing | KW3110 | 51 | 0.6 | ± | 0.1 | 0.7 | ± | 0.1 | 0.9 | ± | 0.1 | 1.3 | ± | 0.1 | 1.4 | ± | 0.1 | 1.3 | ± | 0.1 | 1.0 | ± | 0.1 | 0.1 | | ± | 0.1 | 0.3 | | ± | 0.1 | 0.6 | | ± | 0.1† | 0.8 | | ± | 0.1 | 0.7 | | ± | 0.1 | 0.4 | | ± | 0.1 |
|  | Placebo | 51 | 0.5 | ± | 0.1 | 0.7 | ± | 0.1 | 0.9 | ± | 0.1 | 1.3 | ± | 0.1 | 1.6 | ± | 0.1 | 1.3 | ± | 0.1 | 1.1 | ± | 0.1 | 0.2 | | ± | 0.1 | 0.4 | | ± | 0.1 | 0.8 | | ± | 0.1 | 1.1 | | ± | 0.1 | 0.8 | | ± | 0.1 | 0.6 | | ± | 0.1 |
| Blocked nose | KW3110 | 51 | 0.5 | ± | 0.1 | 0.5 | ± | 0.1 | 0.6 | ± | 0.1 | 0.9 | ± | 0.1 | 1.0 | ± | 0.1 | 1.0 | ± | 0.1 | 0.7 | ± | 0.1 | 0.0 | | ± | 0.1 | 0.1 | | ± | 0.1 | 0.4 | | ± | 0.1 | 0.5 | | ± | 0.1† | 0.5 | | ± | 0.1 | 0.3 | | ± | 0.1 |
|  | Placebo | 51 | 0.4 | ± | 0.1 | 0.5 | ± | 0.1 | 0.6 | ± | 0.1 | 0.9 | ± | 0.1 | 1.2 | ± | 0.1 | 1.0 | ± | 0.1 | 0.7 | ± | 0.1 | 0.1 | | ± | 0.1 | 0.3 | | ± | 0.1 | 0.6 | | ± | 0.1 | 0.8 | | ± | 0.1 | 0.6 | | ± | 0.1 | 0.4 | | ± | 0.1 |
| Itchy nose | KW3110 | 51 | 0.3 | ± | 0.1 | 0.3 | ± | 0.1 | 0.4 | ± | 0.1 | 0.8 | ± | 0.1 | 0.9 | ± | 0.1 | 0.9 | ± | 0.1 | 0.5 | ± | 0.1 | 0.0 | | ± | 0.1 | 0.1 | | ± | 0.1 | 0.6 | | ± | 0.1 | 0.7 | | ± | 0.1 | 0.7 | | ± | 0.2 | 0.3 | | ± | 0.1 |
|  | Placebo | 51 | 0.2 | ± | 0.1 | 0.2 | ± | 0.1 | 0.4 | ± | 0.1 | 0.9 | ± | 0.1 | 1.0 | ± | 0.1 | 0.8 | ± | 0.1 | 0.5 | ± | 0.1 | 0.1 | | ± | 0.1 | 0.2 | | ± | 0.1 | 0.7 | | ± | 0.1 | 0.8 | | ± | 0.1 | 0.7 | | ± | 0.1 | 0.4 | | ± | 0.1 |
| Itchy eyes | KW3110 | 51 | 0.2 | ± | 0.1 | 0.4 | ± | 0.1 | 0.7 | ± | 0.1 | 1.4 | ± | 0.2 | 1.5 | ± | 0.1 | 1.3 | ± | 0.1 | 0.8 | ± | 0.1 | 0.1 | | ± | 0.1 | 0.5 | | ± | 0.1 | 1.1 | | ± | 0.2 | 1.2 | | ± | 0.2 | 1.0 | | ± | 0.2 | 0.6 | | ± | 0.1 |
|  | Placebo | 51 | 0.3 | ± | 0.1 | 0.5 | ± | 0.1 | 0.7 | ± | 0.1 | 1.3 | ± | 0.1 | 1.6 | ± | 0.1 | 1.2 | ± | 0.1 | 0.9 | ± | 0.1 | 0.2 | | ± | 0.1 | 0.4 | | ± | 0.1 | 1.0 | | ± | 0.1 | 1.3 | | ± | 0.2 | 0.9 | | ± | 0.1 | 0.6 | | ± | 0.1 |
| Watery eyes | KW3110 | 51 | 0.1 | ± | 0.0 | 0.2 | ± | 0.1 | 0.3 | ± | 0.1 | 0.8 | ± | 0.1 | 0.7 | ± | 0.1 | 0.7 | ± | 0.1 | 0.3 | ± | 0.1 | 0.0 | | ± | 0.1 | 0.2 | | ± | 0.1 | 0.6 | | ± | 0.1 | 0.6 | | ± | 0.1 | 0.6 | | ± | 0.1 | 0.2 | | ± | 0.1 |
|  | Placebo | 51 | 0.1 | ± | 0.0 | 0.2 | ± | 0.1 | 0.3 | ± | 0.1 | 0.6 | ± | 0.1 | 0.7 | ± | 0.1 | 0.5 | ± | 0.1 | 0.3 | ± | 0.1 | 0.0 | | ± | 0.0 | 0.2 | | ± | 0.1 | 0.5 | | ± | 0.1 | 0.6 | | ± | 0.1 | 0.3 | | ± | 0.1 | 0.2 | | ± | 0.1 |
| Nasal and eye symptoms | KW3110 | 51 | 2.1 | ± | 0.3 | 2.6 | ± | 0.3 | 3.5 | ± | 0.4 | 6.1 | ± | 0.6 | 6.8 | ± | 0.5 | 6.4 | ± | 0.7 | 4.3 | ± | 0.5 | 0.5 | | ± | 0.3 | 1.4 | | ± | 0.4† | 3.9 | | ± | 0.6 | 4.6 | | ± | 0.6 | 4.3 | | ± | 0.8 | 2.2 | | ± | 0.5 |
|  | Placebo | 51 | 2.0 | ± | 0.2 | 2.7 | ± | 0.3 | 3.7 | ± | 0.4 | 6.3 | ± | 0.5 | 7.7 | ± | 0.6 | 6.0 | ± | 0.4 | 4.5 | ± | 0.5 | 0.8 | | ± | 0.2 | 1.8 | | ± | 0.4 | 4.3 | | ± | 0.5 | 5.7 | | ± | 0.6 | 4.0 | | ± | 0.5 | 2.5 | | ± | 0.5 |
| Nasal symptoms | KW3110 | 51 | 1.8 | ± | 0.2 | 2.1 | ± | 0.3 | 2.5 | ± | 0.3 | 3.9 | ± | 0.4 | 4.6 | ± | 0.4 | 4.5 | ± | 0.5 | 3.2 | ± | 0.3 | 0.3 | | ± | 0.3 | 0.7 | | ± | 0.3* | 2.2 | | ± | 0.4* | 2.8 | | ± | 0.4† | 2.7 | | ± | 0.5 | 1.4 | | ± | 0.4† |
|  | Placebo | 51 | 1.5 | ± | 0.2 | 2.1 | ± | 0.2 | 2.7 | ± | 0.2 | 4.3 | ± | 0.3 | 5.4 | ± | 0.4 | 4.3 | ± | 0.3 | 3.3 | ± | 0.3 | 0.6 | | ± | 0.2 | 1.2 | | ± | 0.2 | 2.8 | | ± | 0.3 | 3.8 | | ± | 0.4 | 2.8 | | ± | 0.4 | 1.8 | | ± | 0.3 |
| Eye symptoms | KW3110 | 51 | 0.3 | ± | 0.1 | 0.5 | ± | 0.1 | 1.0 | ± | 0.2 | 2.1 | ± | 0.2 | 2.2 | ± | 0.2 | 1.9 | ± | 0.2 | 1.1 | ± | 0.2 | 0.2 | | ± | 0.1 | 0.7 | | ± | 0.2 | 1.8 | | ± | 0.3 | 1.9 | | ± | 0.2 | 1.6 | | ± | 0.3 | 0.8 | | ± | 0.2 |
|  | Placebo | 51 | 0.5 | ± | 0.1 | 0.6 | ± | 0.1 | 1.0 | ± | 0.2 | 1.9 | ± | 0.2 | 2.3 | ± | 0.2 | 1.6 | ± | 0.2 | 1.2 | ± | 0.2 | 0.2 | | ± | 0.1 | 0.6 | | ± | 0.2 | 1.5 | | ± | 0.2 | 1.9 | | ± | 0.2 | 1.2 | | ± | 0.2 | 0.7 | | ± | 0.2 |
| Daily life | KW3110 | 51 | 0.8 | ± | 0.2 | 1.0 | ± | 0.3 | 1.2 | ± | 0.3 | 2.6 | ± | 0.5 | 2.8 | ± | 0.5 | 2.5 | ± | 0.5 | 1.7 | ± | 0.4 | 0.2 | | ± | 0.2 | 0.4 | | ± | 0.3 | 1.8 | | ± | 0.5 | 2.0 | | ± | 0.4 | 1.8 | | ± | 0.5 | 1.0 | | ± | 0.4 |
|  | Placebo | 51 | 0.5 | ± | 0.2 | 0.9 | ± | 0.2 | 1.0 | ± | 0.2 | 2.5 | ± | 0.4 | 3.0 | ± | 0.5 | 2.0 | ± | 0.4 | 1.3 | ± | 0.3 | 0.4 | | ± | 0.2 | 0.5 | | ± | 0.2 | 2.0 | | ± | 0.4 | 2.5 | | ± | 0.5 | 1.4 | | ± | 0.4 | 0.8 | | ± | 0.4 |
| Out-door activities | KW3110 | 51 | 0.1 | ± | 0.1 | 0.2 | ± | 0.1* | 0.2 | ± | 0.1** | 0.9 | ± | 0.2 | 1.0 | ± | 0.2 | 0.9 | ± | 0.3 | 0.5 | ± | 0.1 | 0.0 | | ± | 0.1 | 0.1 | | ± | 0.1* | 0.8 | | ± | 0.2 | 0.9 | | ± | 0.2 | 0.8 | | ± | 0.3 | 0.4 | | ± | 0.1 |
|  | Placebo | 51 | 0.2 | ± | 0.1 | 0.3 | ± | 0.1 | 0.5 | ± | 0.1 | 1.3 | ± | 0.2 | 1.3 | ± | 0.2 | 0.9 | ± | 0.2 | 0.5 | ± | 0.1 | 0.1 | | ± | 0.1 | 0.2 | | ± | 0.1 | 1.0 | | ± | 0.3 | 1.0 | | ± | 0.2 | 0.7 | | ± | 0.2 | 0.3 | | ± | 0.2 |
| Social functioning | KW3110 | 51 | 0.3 | ± | 0.1 | 0.5 | ± | 0.2 | 0.5 | ± | 0.2 | 0.9 | ± | 0.2 | 1.1 | ± | 0.3 | 1.1 | ± | 0.3 | 0.7 | ± | 0.2 | 0.2 | | ± | 0.2 | 0.2 | | ± | 0.1 | 0.6 | | ± | 0.2 | 0.8 | | ± | 0.2 | 0.8 | | ± | 0.3 | 0.5 | | ± | 0.2 |
|  | Placebo | 51 | 0.3 | ± | 0.1 | 0.3 | ± | 0.1 | 0.4 | ± | 0.1 | 1.1 | ± | 0.2 | 1.2 | ± | 0.2 | 1.1 | ± | 0.2 | 0.6 | ± | 0.2 | 0.0 | | ± | 0.1 | 0.2 | | ± | 0.1 | 0.9 | | ± | 0.2 | 1.0 | | ± | 0.2 | 0.8 | | ± | 0.2 | 0.3 | | ± | 0.2 |
| Sleep disurbance | KW3110 | 51 | 0.1 | ± | 0.1 | 0.2 | ± | 0.1 | 0.1 | ± | 0.1 | 0.5 | ± | 0.1 | 0.5 | ± | 0.1 | 0.4 | ± | 0.1 | 0.3 | ± | 0.1 | 0.0 | | ± | 0.0 | 0.0 | | ± | 0.1 | 0.4 | | ± | 0.1 | 0.4 | | ± | 0.1 | 0.3 | | ± | 0.1 | 0.1 | | ± | 0.1 |
|  | Placebo | 51 | 0.2 | ± | 0.1 | 0.2 | ± | 0.1 | 0.2 | ± | 0.1 | 0.5 | ± | 0.1 | 0.5 | ± | 0.1 | 0.3 | ± | 0.1 | 0.2 | ± | 0.1 | 0.0 | | ± | 0.1 | 0.0 | | ± | 0.1 | 0.2 | | ± | 0.1 | 0.3 | | ± | 0.1 | 0.1 | | ± | 0.1 | 0.0 | | ± | 0.1 |
| Physical problems | KW3110 | 51 | 0.4 | ± | 0.1 | 0.5 | ± | 0.1 | 0.5 | ± | 0.2 | 1.1 | ± | 0.2 | 1.0 | ± | 0.2 | 0.8 | ± | 0.2 | 0.7 | ± | 0.2 | 0.1 | | ± | 0.1 | 0.1 | | ± | 0.2 | 0.7 | | ± | 0.2 | 0.7 | | ± | 0.2 | 0.5 | | ± | 0.2 | 0.3 | | ± | 0.2 |
|  | Placebo | 51 | 0.3 | ± | 0.1 | 0.4 | ± | 0.1 | 0.5 | ± | 0.1 | 1.0 | ± | 0.2 | 1.3 | ± | 0.2 | 1.0 | ± | 0.2 | 0.7 | ± | 0.2 | 0.2 | | ± | 0.1 | 0.2 | | ± | 0.1 | 0.7 | | ± | 0.2 | 1.0 | | ± | 0.2 | 0.7 | | ± | 0.2 | 0.5 | | ± | 0.1 |
| Emotinal function | KW3110 | 51 | 0.5 | ± | 0.2 | 0.4 | ± | 0.1 | 0.8 | ± | 0.3 | 1.8 | ± | 0.4 | 1.7 | ± | 0.4 | 1.6 | ± | 0.4 | 1.2 | ± | 0.3 | -0.1 | | ± | 0.1† | 0.3 | | ± | 0.3 | 1.3 | | ± | 0.4 | 1.2 | | ± | 0.4 | 1.1 | | ± | 0.4 | 0.7 | | ± | 0.3 |
|  | Placebo | 51 | 0.2 | ± | 0.1 | 0.5 | ± | 0.1 | 0.5 | ± | 0.2 | 1.6 | ± | 0.4 | 1.7 | ± | 0.4 | 1.2 | ± | 0.3 | 0.8 | ± | 0.2 | 0.2 | | ± | 0.1 | 0.3 | | ± | 0.2 | 1.4 | | ± | 0.3 | 1.4 | | ± | 0.4 | 1.0 | | ± | 0.3 | 0.6 | | ± | 0.2 |
| QOL-related questionnaire | KW3110 | 51 | 2.2 | ± | 0.6 | 2.6 | ± | 0.7 | 3.4 | ± | 1.0 | 7.8 | ± | 1.6 | 8.1 | ± | 1.5 | 7.4 | ± | 1.6 | 5.2 | ± | 1.2 | 0.4 | | ± | 0.7† | 1.2 | | ± | 0.9 | 5.6 | | ± | 1.6† | 6.0 | | ± | 1.4 | 5.3 | | ± | 1.5 | 3.0 | | ± | 1.2 |
|  | Placebo | 51 | 1.7 | ± | 0.5 | 2.7 | ± | 0.6 | 3.2 | ± | 0.7 | 8.0 | ± | 1.4 | 9.0 | ± | 1.5 | 6.5 | ± | 1.2 | 4.1 | ± | 0.9 | 0.9 | | ± | 0.5 | 1.5 | | ± | 0.6 | 6.3 | | ± | 1.3 | 7.2 | | ± | 1.4 | 4.8 | | ± | 1.2 | 2.4 | | ± | 0.9 |
| Overall face scale | KW3110 | 51 | 1.1 | ± | 0.1 | 1.1 | ± | 0.1 | 1.3 | ± | 0.1 | 1.6 | ± | 0.1 | 1.8 | ± | 0.1 | 1.6 | ± | 0.1 | 1.3 | ± | 0.1 | 0.0 | | ± | 0.1 | 0.2 | | ± | 0.1 | 0.5 | | ± | 0.1 | 0.7 | | ± | 0.1 | 0.5 | | ± | 0.1 | 0.2 | | ± | 0.1 |
|  | Placebo | 51 | 1.3 | ± | 0.1 | 1.3 | ± | 0.1 | 1.4 | ± | 0.1 | 1.8 | ± | 0.1 | 2.0 | ± | 0.1 | 1.7 | ± | 0.1 | 1.4 | ± | 0.1 | 0.0 | | ± | 0.1 | 0.1 | | ± | 0.1 | 0.5 | | ± | 0.1 | 0.7 | | ± | 0.1 | 0.4 | | ± | 0.1 | 0.1 | | ± | 0.1 |

Mean ± standard error.

†p < 0.10, *p < 0.05, **p < 0.01 (the Mann–Whitney U test between the two groups).

"Amount of change in score" is the score of difference from 0W.

**Table S9.** Classification of severity of allergic rhinitis symptoms by otorhinolaryngology (subjects who did not use medication).

|  |  |  | Score | | | | | | | | | | | | Amount of change in score | | | | | | | | |
| --- | --- | --- | --- | --- | --- | --- | --- | --- | --- | --- | --- | --- | --- | --- | --- | --- | --- | --- | --- | --- | --- | --- | --- |
| Parameter | Group | n | 0W | | | 4W | | | 8W | | | 12W | | | 4W | | | 8W | | | 12W | | |
| Paroxysmal sneezing or rhinorrhea | KW3110 | 51 | 0.9 | ± | 0.1 | 1.0 | ± | 0.1† | 1.4 | ± | 0.1 | 0.9 | ± | 0.1 | 0.1 | ± | 0.1* | 0.6 | ± | 0.1 | 0.1 | ± | 0.1 |
|  | Placebo | 51 | 0.9 | ± | 0.1 | 1.3 | ± | 0.1 | 1.7 | ± | 0.1 | 1.0 | ± | 0.1 | 0.4 | ± | 0.1 | 0.8 | ± | 0.1 | 0.1 | ± | 0.1 |
| Nasal blockage | KW3110 | 51 | 0.5 | ± | 0.1 | 0.5 | ± | 0.1 | 0.9 | ± | 0.1 | 0.5 | ± | 0.1 | 0.1 | ± | 0.1 | 0.5 | ± | 0.1 | 0.0 | ± | 0.1 |
|  | Placebo | 51 | 0.5 | ± | 0.1 | 0.6 | ± | 0.1 | 1.1 | ± | 0.1 | 0.5 | ± | 0.1 | 0.2 | ± | 0.1 | 0.5 | ± | 0.1 | 0.0 | ± | 0.1 |
| Classification of severity of allergic rhinitis symptoms | KW3110 | 51 | 0.9 | ± | 0.1 | 1.0 | ± | 0.1* | 1.5 | ± | 0.1 | 1.0 | ± | 0.1 | 0.1 | ± | 0.1* | 0.6 | ± | 0.1 | 0.1 | ± | 0.1 |
|  | Placebo | 51 | 0.9 | ± | 0.1 | 1.3 | ± | 0.1 | 1.7 | ± | 0.1 | 1.1 | ± | 0.1 | 0.4 | ± | 0.1 | 0.8 | ± | 0.2 | 0.2 | ± | 0.1 |

Mean ± standard error.

†p < 0.10, *p < 0.05 (the Mann–Whitney U test between the two groups).

"Amount of change in score" is the score of difference from 0W.

**Table S10.** Scores of Nasal and Eye Symptoms Questionnaire by diary (subjects who did not use medication).

|  |  |  | Score | | | | | | | | | | | | | | | | | | Amount of change in score | | | | | | | | | | | | | | |
| --- | --- | --- | --- | --- | --- | --- | --- | --- | --- | --- | --- | --- | --- | --- | --- | --- | --- | --- | --- | --- | --- | --- | --- | --- | --- | --- | --- | --- | --- | --- | --- | --- | --- | --- | --- |
| Parameter | Group | n | 2W | | | 4W | | | 6W | | | 8W | | | 10W | | | 12W | | | 4W | | | 6W | | | 8W | | | 10W | | | 12W | | |
| Sneezing | KW3110 | 51 | 0.54 | ± | 0.06 | 0.68 | ± | 0.07 | 0.94 | ± | 0.08 | 1.10 | ± | 0.10 | 1.03 | ± | 0.10 | 0.90 | ± | 0.10 | 0.14 | ± | 0.05 | 0.40 | ± | 0.08 | 0.57 | ± | 0.09 | 0.49 | ± | 0.09 | 0.37 | ± | 0.10 |
|  | Placebo | 51 | 0.56 | ± | 0.07 | 0.67 | ± | 0.08 | 0.99 | ± | 0.07 | 1.27 | ± | 0.09 | 1.22 | ± | 0.10 | 1.02 | ± | 0.09 | 0.11 | ± | 0.04 | 0.43 | ± | 0.05 | 0.72 | ± | 0.09 | 0.66 | ± | 0.09 | 0.46 | ± | 0.08 |
| Runny nose | KW3110 | 51 | 0.70 | ± | 0.07 | 0.74 | ± | 0.08 | 0.92 | ± | 0.09 | 1.08 | ± | 0.10* | 1.06 | ± | 0.10* | 0.88 | ± | 0.10 | 0.04 | ± | 0.06 | 0.22 | ± | 0.09 | 0.38 | ± | 0.10* | 0.36 | ± | 0.11* | 0.18 | ± | 0.10† |
|  | Placebo | 51 | 0.69 | ± | 0.08 | 0.77 | ± | 0.08 | 1.04 | ± | 0.09 | 1.44 | ± | 0.11 | 1.35 | ± | 0.11 | 1.07 | ± | 0.10 | 0.08 | ± | 0.05 | 0.35 | ± | 0.07 | 0.74 | ± | 0.10 | 0.66 | ± | 0.10 | 0.38 | ± | 0.09 |
| Blocked nose | KW3110 | 51 | 0.34 | ± | 0.06 | 0.44 | ± | 0.07 | 0.52 | ± | 0.08 | 0.68 | ± | 0.09 | 0.65 | ± | 0.09 | 0.50 | ± | 0.08 | 0.10 | ± | 0.05 | 0.19 | ± | 0.08 | 0.35 | ± | 0.10* | 0.31 | ± | 0.10 | 0.16 | ± | 0.09 |
|  | Placebo | 51 | 0.27 | ± | 0.07 | 0.38 | ± | 0.07 | 0.57 | ± | 0.07 | 0.90 | ± | 0.10 | 0.77 | ± | 0.10 | 0.59 | ± | 0.09 | 0.10 | ± | 0.03 | 0.30 | ± | 0.07 | 0.62 | ± | 0.09 | 0.50 | ± | 0.10 | 0.32 | ± | 0.09 |
| Itcy eyes | KW3110 | 51 | 0.21 | ± | 0.06 | 0.38 | ± | 0.08 | 0.91 | ± | 0.12 | 1.14 | ± | 0.12 | 1.00 | ± | 0.10 | 0.65 | ± | 0.11 | 0.17 | ± | 0.05 | 0.70 | ± | 0.12 | 0.92 | ± | 0.13 | 0.79 | ± | 0.11 | 0.44 | ± | 0.11 |
|  | Placebo | 51 | 0.25 | ± | 0.06 | 0.38 | ± | 0.08 | 0.84 | ± | 0.09 | 1.28 | ± | 0.11 | 1.03 | ± | 0.12 | 0.75 | ± | 0.10 | 0.13 | ± | 0.04 | 0.58 | ± | 0.07 | 1.03 | ± | 0.11 | 0.77 | ± | 0.12 | 0.50 | ± | 0.10 |
| Watery eyes | KW3110 | 51 | 0.09 | ± | 0.03 | 0.17 | ± | 0.05 | 0.43 | ± | 0.08 | 0.54 | ± | 0.09 | 0.46 | ± | 0.10 | 0.30 | ± | 0.08 | 0.07 | ± | 0.04 | 0.33 | ± | 0.08 | 0.44 | ± | 0.09 | 0.36 | ± | 0.09 | 0.21 | ± | 0.07 |
|  | Placebo | 51 | 0.12 | ± | 0.04 | 0.18 | ± | 0.05 | 0.40 | ± | 0.07 | 0.53 | ± | 0.09 | 0.41 | ± | 0.09 | 0.28 | ± | 0.07 | 0.06 | ± | 0.03 | 0.28 | ± | 0.05 | 0.41 | ± | 0.07 | 0.28 | ± | 0.08 | 0.15 | ± | 0.06 |

Mean ± standard error.

†p < 0.10, *p < 0.05 (the Mann–Whitney U test between the two groups).

"Amount of change in score" is the score of difference from 0W.
